# TANGO: effect of tango Argentino on cancer-associated fatigue in breast cancer patients—study protocol for a randomized controlled trial

**DOI:** 10.1186/s13063-021-05869-3

**Published:** 2021-12-02

**Authors:** Shiao Li Oei, Thomas Rieser, Sarah Becker, Jessica Groß, Harald Matthes, Friedemann Schad, Anja Thronicke

**Affiliations:** 1 Research Institute Havelhöhe at the Hospital Gemeinschaftskrankenhaus Havelhöhe, Berlin, Germany; 2 Institute for Social Medicine, Epidemiology and Health Economics, Charité – Universitätsmedizin Berlin, Freie Universität Berlin, Humboldt-Universität zu Berlin, Berlin Institute of Health, Berlin, Germany; 3 Breast Cancer Centre, Hospital Gemeinschaftskrankenhaus Havelhöhe, Berlin, Germany; 4 Medical Clinic for Gastroenterology, Infectiology and Rheumatology CBF Charité – Universitätsmedizin Berlin, Freie Universität Berlin, Humboldt-Universität zu Berlin, Berlin Institute of Health, Berlin, Germany; 5 Institute for Gastroenterology, Hospital Gemeinschaftskrankenhaus Havelhöhe, Berlin, Germany; 6 Interdisciplinary Oncology and Palliative Care, Hospital Gemeinschaftskrankenhaus Havelhöhe, Berlin, Germany

**Keywords:** Breast cancer, Dance, Fatigue, Insomnia, Health-related quality of life

## Abstract

**Background:**

The majority of breast cancer patients suffer from persistent impairments after completion of their primary oncological therapy. Cancer-related fatigue (CRF) in particular is a multidimensional syndrome having a profound negative impact on the quality of life. To counter CRF symptoms, physical activities are suggested as first-line interventions, mind-body therapies have been shown to be effective, and music therapy can also reduce anxiety and stress in breast cancer patients. Tango therapy that combines various elements can have an impact on physical, psychological, and cognitive abilities and could therefore have a beneficial effect on breast cancer patients. The purpose of this study is to investigate whether a 6-week tango module is suited as a therapeutic approach for people after primary breast cancer therapy to favorably influence their quality of life, especially CRF levels.

**Methods:**

Sixty patients with a diagnosis for stage I–III breast cancer 12–48 months before enrollment and with CRF (age > 18) will be recruited and randomized 1:1 to a tango or a waiting-list group. Movement concepts using elements of Argentine tango (self-awareness, musical and spatial perception, self-perception, playfulness, shared experience) will be examined with the participants during six consecutive weekly 1-h tango sessions. The primary outcome will be the improvement of CRF (German version of the Cancer Fatigue Scale), and the secondary outcomes will be the improvement in sleep quality (Pittsburgh Sleep Quality Index) and quality of life (EORTC-QLQ-C30). Patient-reported outcomes will be measured at baseline and 6 weeks later; follow-up will be performed 6, 12, and 24 months after baseline. An evaluation will be performed by means of descriptive data analyses.

**Discussion:**

Argentine tango, as a music-based movement therapy, can influence different skills and may improve several outcomes. The therapeutic use of Argentine tango in the care of breast cancer patients has not yet been reported. It is anticipated that participants receiving the tango module will have improved CRF, sleep, and quality of life scores compared to a waitlist control.

**Trial registration:**

German Clinical Trials Registry (DRKS) DRKS00021601. Retrospectively registered on 21 August 2020

## Administrative information

Note: The numbers in curly brackets in this protocol refer to SPIRIT checklist item numbers. The order of the items has been modified to group similar items (see http://www.equator-network.org/reporting-guidelines/spirit-2727-statement-defining-standard-protocol-items-for-clinical-trials/).

**Table:** 

Title {1}	TANGO - Effect of Tango Argentino on cancer-associated fatigue in breast cancer patients: Study Protocol for a Randomized Controlled Trial
Trial registration {2a and 2b}.	Trial registration number DRKS00021601. Retrospectively registered on 21 August 2020.
Protocol version {3}	The current protocol version is version 2.0, dated from 23 March 2020.
Funding {4}	The primary sponsor is the Forschungsinstitut Havelhöhe (FIH) gGmbH am Gemeinschaftskrankenhaus Havelhöhe.
Author details {5a}	SLO, MH, FS, AT: Research Institute Havelhöhe gGmbH at Hospital Gemeinschaftskrankenhaus Havelhöhe, Berlin, Germany; TR, HM, AT: Charité – Universitätsmedizin Berlin, corporate member of Freie Universität Berlin and Humboldt-Universität zu Berlin, Institute of Social Medicine, Epidemiology and Health Economics, Berlin, Germany; SB, JG: Hospital Gemeinschaftskrankenhaus Havelhöhe Breast Cancer Centre, Berlin, Germany; MH: Medical Clinic for Gastroenterology, Infectiology and Rheumatology CBF, Charité – Universitätsmedizin Berlin, corporate member of Freie Universität Berlin and Humboldt-Universität zu Berlin, MH, FS: Hospital Gemeinschaftskrankenhaus Havelhöhe, Interdisciplinary Oncology and Palliative Care, Berlin
Name and contact information for the trial sponsor {5b}	Research Institute Havelhöhe gGmbH at Hospital Gemeinschaftskrankenhaus Havelhöhe, Berlin, Germany Trial’s principal investigator: Friedemann Schad, fschad@havelhoehe.de; scientific coordinator: Anja Thronicke, anja.thronicke@havelhoehe.de
Role of sponsor {5c}	The sponsor is non-commercial. The sponsor ensures quality management, qualified and trained personnel, study protocol compliance, submission of relevant study documents to the ethics committee and regulatory authorities. He supports the study with trial unit facilities and study nurses.

## Introduction

### Background and rationale {6a}

Breast cancer still is the most common cancer in women worldwide [1]. About one-third of breast cancer patients experience moderate to severe fatigue symptoms [2, 3]. Cancer-related fatigue (CRF) is a multidimensional syndrome having a profound negative impact on health-related quality of life (HRQL) and is a very prevalent and distressing side effect among breast cancer patients, which may often persist for many years after oncological treatment [2, 4]. In a systematic review, it was found that an ameliorative benefit on HRQL can be reached with physical activity interventions such as yoga, physical self-management, complementary exercises, art therapies, mind-body exercise therapies, and cognitive-behavioral therapies [5]. Currently, there is convincing evidence that specific doses of aerobic and/or resistance training could improve common cancer-related health outcomes, including fatigue, and it is recommended that physical activities are proposed as a first-line intervention for improving HRQL and counteracting CRF symptoms [6, 7]. Mindfulness-based interventions, such as meditation and yoga, in particular, have been shown to be effective in reducing CRF levels [8]. Unfortunately, a lack of motivation and fatigue and a lack of time are often barriers to physical activity [9]. Several results from systematic reviews reveal mindfulness-based art therapy improves anxiety, depression, fatigue, stress, and HRQL [10–12]. Especially, cancer-related cognitive complaints such as fatigue, insomnia, and psychological distress might be managed through multimodal approaches. Another form of exercise therapy, which includes cognitive, emotional, and volitional elements, is eurythmy therapy [13], and interestingly, a multicenter study of 126 breast cancer patients reported that a multimodal approach that included eurythmy therapy and psychoeducation resulted in an improvement in HRQL and a significant reduction in symptoms of fatigue [14, 15]. Also, behavioral techniques appear to play an important role in influencing psychosocial functioning and cognitive impairments [16]. Data of a systematic review found a positive role of life review on spiritual well-being, overall distress, and HRQL in patients with terminal or advanced cancer [17], which is important not only at the end of life. For instance, we reported that in breast cancer patients, elaborate consultations and life reviews conducted at first diagnosis were associated with a significant reduction in fatigue symptoms and relevant improvements in HRQL [18, 19]. Music therapy is another suitable creative arts treatment for improving psychological and physical outcomes in cancer patients, and a beneficial effect on anxiety and especially a moderate effect on fatigue and HRQL were found [20]. Hence, dance as a combination of physical activity, music, and mindful elements might be an appropriate and effective approach, to address CRF and to improve HRQL [21]. Protocols for randomized controlled trials of dance therapies for breast cancer patients have been published, to investigate different and various aspects. For example, it has been investigated whether dance therapy can relieve symptoms and stress in breast cancer patients, during radiation therapy [22], and whether traditional Greek dance [23] or belly dance [24] shows physical or psychological benefits. Furthermore, a pilot trial of 31 female cancer survivors and their partners examined ballroom dance, to improve their HRQL [25]. With a dance program for patients with breast cancer across five European countries, positive changes on HRQL and improvements of emotional and social scales were observed [26], and in a feasibility study, the efficacy of a balance training program using elements of Argentine tango for cancer survivors was assessed [27]. Argentine tango is a music-based movement therapy which can influence physical, psychological, and cognitive skills. In addition, it meets women’s desire for social interaction [28]. Tango as a dance form has the advantage that it can be danced improvisationally in a variety of ways: without former experience, in changing roles, paired or in groups, as well as with or without physical contact. Interestingly, a systematic review revealed that Argentine tango has a tendency for positive effects on fatigue and HRQL in Parkinson’s disease patients [29]. To date, no results of high-quality randomized controlled trials have been published on the effectiveness of dance therapies for cancer survivors. The aim of this study is to investigate whether therapeutic tango can improve self-reported quality of life, particularly fatigue levels, in breast cancer survivors.

### Objectives {7}

This study protocol describes a randomized controlled trial for patients with breast cancer and increased CRF receiving either Argentine tango in a tango group or no Argentine tango in a waiting-list control group. The primary outcome measure is the change of CRF levels after 6 weeks. (A) The CRF changes of patients of the tango group will be compared to the waiting-list control group, and (B) prior to and post the 6-week Argentine tango treatment, the CRF changes are evaluated for the whole study cohort. The primary hypothesis is that a module consisting of six consecutive weekly 1-h Argentine tango lessons will promote a significant reduction of fatigue symptoms. Secondary objectives are the improvement of self-reported sleep quality and HRQL, prior to and post the Argentine tango treatment, and to explore the effects after 6, 12, and 24 months follow-up.

### Trial design {8}

**Fig. 1:**
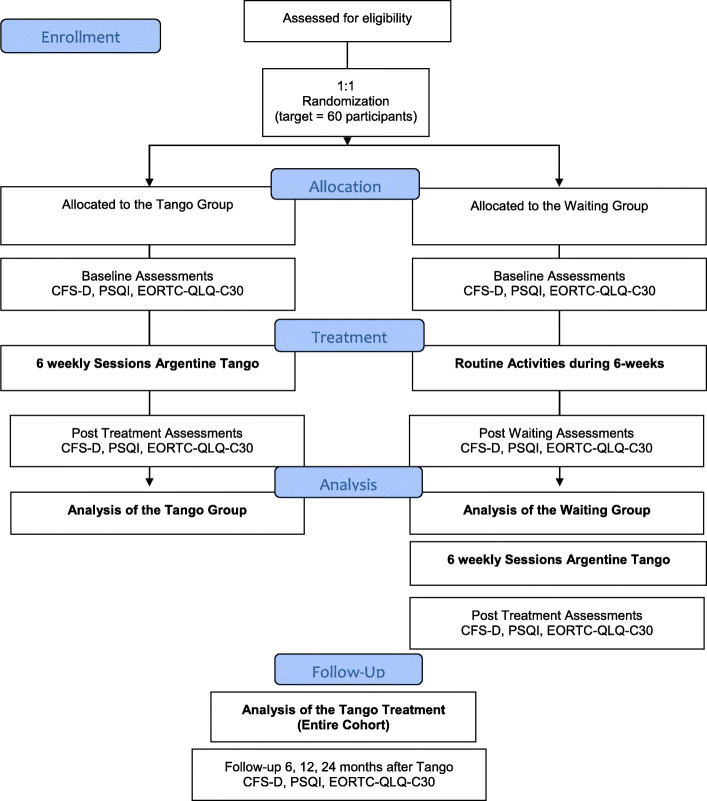
Flow diagram of the study participants according to the Consolidated Standards of Reporting Trials (CONSORT 2010), illustrating the participant’s timeline

This is a single-center, prospective, two-arm randomized controlled trial designed to assess the effects of Argentine tango for people with breast cancer and CRF. The participants will be randomized to either the tango group or the waiting-list control group (Fig. 1). Evaluation of the patient-reported outcomes CRF, sleep quality, and HRQL will be performed.

## Methods: participants, interventions, and outcomes

### Study setting {9}

This study is conducted by the Forschungsinstitut Havelhöhe (FIH) at the hospital Gemeinschaftskrankenhaus Havelhöhe in Berlin (GKHB). Participants are recruited at the primary care facility of the Breast Cancer Centre at the GKHB.

### Eligibility criteria {10}

#### Inclusion, exclusion, and dropout criteria for participants

Participants will be eligible to join the study if they had been diagnosed with breast cancer, have increased levels of fatigue, are willing to participate in this study, and if they meet all following criteria:

- Signed written informed consent
- Age ≥ 18 years
- Stage I–III breast cancer has been diagnosed 12–48 months before enrollment
- Increased levels of fatigue (≥ 12 points in the CFS-D questionnaire)
- Eastern Cooperative Oncology Group (ECOG) performance status 0–2

Patients will not be eligible for the study if they meet one or more of the following exclusion criteria:

- No fatigue at all (≤ 12 points in the CFS-D questionnaire)
- Pregnancy or breastfeeding
- Occurrence of recurrences or metastases
- Concurrent participation in other clinical trials
- Linguistic, medical, psychiatric, cognitive, or other conditions that may compromise the patient’s ability to understand the patient information, comply with the study protocol, or complete the study

Participants must attend at least four of the six tango sessions and answer the questionnaires; if they do not, they will be considered dropouts.

### Who will take informed consent? {26a}

It is the responsibility of the study physician to provide sufficient verbal and written information on the study’s purpose and procedures, information on data protection procedures, possible advantages and disadvantages of participation, and option to withdraw from the study at any time and without any given reason and to take informed consent. Written informed consent will be obtained from all participants prior to study enrollment.

### Additional consent provisions for collection and use of participant data and biological specimens {26b}

The consent form asks whether participants agree that the data collected may be shared and published in a pseudonymized form for research purposes.

### Interventions

#### Explanation for the choice of comparators {6b}

The comparator is a waiting-list control group. All participants will be randomized to either the tango group or the waiting group. The participants of the waiting group will continue routine activities during the 6-week waiting period. During follow-up, patients from the waiting-list control group will receive the same Argentine tango module as the participants of the tango group. The follow-up assessments for both trial arms will be the same (Fig. 1).

#### Intervention description {11a}

The Argentine tango program consists of six weekly 1-h tango sessions, which are conducted by a professional tango teacher and will be carried out in small groups of three to eight participants. Elements of movement and music of Argentine tango will be presented. The coordination, rhythm, and body awareness will be stimulated with harmonic movements to the tango music. Introductory exercises are practiced first without musical accompaniment and then with Argentine tango music. The 6-week program comprises breathing and relaxation exercises, balance finding, and includes tango walking and dancing elements as well as social interaction. The therapist will give explanations and lead guided exercises which are oriented towards walking to music, self-perceptions, and spatial perceptions as outlined in Table 1. Each group session will be structured in a warm-up, main part, and focused ending. The warm-up consists of a standing exercise with the feet open, with the imagination of moving through the body from the toes to the scalp and bringing awareness to the bodily sensations. The main parts have different focuses throughout the six sessions and include theoretical and practical instructions of walking and dancing techniques, repetitions with individual breaks, and explanations as needed (for details see Table 1). Each session ends with a 3-min standing exercise without movement, in silence with feet open and eyes closed.

**Table 1:** Six-week Argentine tango protocol for people with breast cancer and fatigue

Session	Description of the activities
1	***Self-awareness*.** Warming-up (standing exercise, open feet, imaging of moving through the body from toes to scalp bringing awareness to patient’s bodily sensations); axis and muscle tone; tension and relaxation; introduction of the tango position with feet closed, reaching in six directions (floor, sky, front, back, left, right); walking techniques, steps in four directions (forward, backward, left, right); walking forward in the line of dance with music; walking backward in the line of dance with music; changing directions in the line of dance from forward to backward while walking with music; and focused ending (standing exercise in silence with open feet and eyes closed, no movement)
2	***Spatial and musical perception*.** Warming-up; walking technique, steps in four directions; walking in the line of dance to the music; changing directions in the line of dance from forward to backward while walking with music; musicality: double-beat, single-beat, and half-beat; focused ending
3	***Social Perception.*** Warming-up; walking technique, steps in four directions; walking in the line of dance to the music; changing directions in the line of dance from forward to backward while walking with music; musicality: double-beat, single-beat, and half-beat; partner exercises “mirror dance” = no physical contact, distance 2 m; mirror dance in a circle: one person leads the whole group; mirror dance in couples with changing partners walking in squares and walking in the line of dance with music; and focused ending
4	***Playfulness*.** Warming-up; introduction of the “pivot” = turning on one foot; combining walking and pivot; mirror dance (distance 2 m, no physical contact) in couples with changing partners with playful variations of walking together in the line of dance with music and with pivots; and focused ending
5	***Sensation*.** Warming-up; mirror dance in couples (distance 2 m, no physical contact) with changing partners with the elements of the previous four sessions; focus on the quality of movement and connection = not how we move matters, but how we feel moving; and focused ending
6	***Shared experience*.** Warming-up; dancing summary of the five sessions; focus on communal dance experience (distance 2 m, no physical contact); combining oneself with the group; and focused ending

#### Criteria for discontinuing or modifying allocated interventions {11b}

Participants are encouraged to report any discomfort and to perform only movements that do not cause them any problems. If a participant does not attend a session, she is contacted by phone and asked how she is feeling, and options are sought to continue her tango sessions. Adapting to the current COVID-19 pandemic conditions, the tango sessions are conducted in compliance with the national security and hygienic regulations, i.e., all dance movements, including paired actions, will be performed at a distance of 2 m to each other, the tango sessions will be conducted in a large therapy room with adequate oxygen ventilation, and the patients as well as the teacher are wearing surgical masks during the session. Before entering the therapy room, the teacher and the patients have to disinfect their hands with adequate disinfection detergent (including > 75% ethanol). Protective policies are constantly adapted to the locally applicable regulations; currently, participants must be vaccinated or have a valid negative SARS-CoV2 test to participate in Tango sessions.

#### Strategies to improve adherence to interventions {11c}

At the beginning of each session, participants are asked about their feelings with the previous session and experiences, and their preferences are discussed in the group. In case of any problems with adhering to the tango lesson, ways will be sought to carry out at least minimal essential parts of the treatment. The number of sessions each participant actually attended will be recorded. For the follow-up surveys, questionnaires will be sent with prepaid return envelopes, and if necessary, participants will be reminded by phone call to fill in and return them.

#### Relevant concomitant care permitted or prohibited during the trial {11d}

Concomitant medications including nutritional supplements, vitamins, and natural remedies will be registered in the case report form. Standard routine care and drugs as endocrine treatments do not affect patients’ eligibility to enter or patients’ participation during the trial.

#### Provisions for post-trial care {30}

After the whole tango module, participants will be provided with information about appropriate online tango exercises and classes that they could attend on a self-pay basis.

### Outcomes {12}

Outcome measures are 1. the change of CRF levels, 2. the change of sleep quality, and 3. the change of HRQL.

1. CRF levels will be investigated with the German version of the certified and validated Cancer Fatigue Scale (CFS-D) [30]. It consists of a 15-item questionnaire on three subscales (physical, cognitive, and affective fatigue), based on a 5-point Likert scale with a possible range of 0 (no fatigue) to 60 (maximum fatigue) [30]. The CFS-D is highly reliable with a robust validity and classifies values ≥ 30 points as clear symptoms of fatigue, ≥ 24 points as suspected moderate fatigue, and ≤ 23 points as only minor or no fatigue symptoms. A decline of scores indicates an improvement of fatigue while higher scores represent a higher degree of fatigue symptoms.
2. Self-reported sleep quality will be evaluated by the certified and validated Pittsburgh Sleep Quality Index (PSQI) questionnaire [31]. This questionnaire is composed of seven sleep-related subscales (subjective quality, latency, duration, habitual efficiency, disturbances, use of sleeping medication, and daytime sleepiness) all scored between 0 and 3. The global PSQI sum scores range from 0 (no sleep disturbance) to 21 (maximally disturbed sleep), while a PSQI score greater than 5 classifies poor sleepers.
3. Self-reported HRQL will be investigated with the European Organization for Research and Treatment of Cancer Questionnaire C30 (EORTC-QLQ-C30) [32], which is structured into 15 different subscales (1 global health, 5 functional, and 9 symptoms, including fatigue and insomnia scales). Equations will be made as described in the EORTC-QLQ-C30 manual. The EORTC scores range from 0 to 100, and higher scores represent a better self-reported level in the functional dimensions but a higher degree of symptom burden.

*Baseline information* on diagnosis, such as histology, tumor classification, and potential previous treatments, will be collected from the clinical interview at study inclusion and the participants’ medical records. All participants will be asked to answer all questionnaires at baseline, prior to and post-treatment, or waiting, and likewise in the framework of mid- and long-term surveys, 6, 12, and 24 months after completing the tango module (Fig. 1). Follow-up surveys will also ask about interim experiences with dance and movements, what medications were taken, and whether there are interests in continuing and online interventions. The courses of fatigue, sleep quality, and HRQL of participants of this study will be compared to those of previous patients of the GKHB Breast Cancer Center [18, 33].

### Participant timeline {13}

In Fig. 1, trial procedures from enrollment to the end of the trial are illustrated.

### Sample size {14}

The expected longitudinal differences between the means of the outcomes assessed will be estimated based on our previously published breast cancer studies [18, 33]. Previously, significant associations with medium effect sizes between non-pharmacological interventions and an improvement in fatigue were observed, and we found a mean difference of 7–12% [33]. For the characterization of the group and longitudinal differences, Student’s *t*-tests and Pearson’s chi-squared tests with Yates’ continuity correction were performed. For the calculation of the sample size, medium to large effect size differences (Cohen’s *d* of 0.8) and a power of 80%, as well as a significant level of 5% (two-sided) for the primary outcome fatigue, are assumed. Thus, a case number of 25 patients per group will be necessary. Assuming a dropout rate of 20%, we need to recruit and randomize 60 participants.

### Recruitment {15}

Breast cancer patients who have been treated at the GKH Breast Cancer Center and who have given their consent to be contacted for clinical studies will be briefly screened for eligibility, contacted by phone call, and invited to participate in this study. A clinical interview is scheduled with interested and eligible patients. After obtaining written informed consent and after the patient’s enrollment in this study, sociodemographic and clinical data (age, body mass index, tumor stage, hormonal status, concomitant medications, sports activities) are surveyed, and questionnaires will be given to participants.

### Assignment of interventions: allocation

#### Sequence generation {16a}

The randomization sequence has been created [34] using the website www.jerrydallal.com/random/random_block_size_r.htm with a 1:1 allocation, using block sizes of four to six. The randomization plan for subjects no. 1–40 was generated on 12 May 2020 (Seed No. 15277) and for subjects no. 41–60 on 2 March 2021 (Seed No. 15434).

#### Concealment mechanism {16b}

In the temporal order of the study inclusion, allocation will be made according to the randomization plan to either the tango or the waiting group. Participants, physicians, and therapists are blinded to the randomization and allocation processes and will have no influence on the assignment of participants to each of both groups.

#### Implementation {16c}

In a clinical interview, a study physician collects patient clinical and demographic data and provides information about the study. All patients who give their written consent to participate in the study will be enrolled in this study. Unique identifiers were generated using an ID-generator software [35]. A study investigator will register the participants, using the randomization plan to allocate to either the tango or waiting group. According to the availability to participate in a tango course, the participants are asked to complete and return the questionnaires at specified dates. The flow diagram of the study design is depicted in Fig. 1.

All patient data is handled in accordance with the General Data Protection Regulation (GDPR). All data from the participants will be maintained confidentially before, during, and after the trial and is stored securely at the study site, with limited access.

### Assignment of interventions: blinding

#### Who will be blinded {17a}

All participants, physicians, and therapists are blinded to the randomization and allocation process. Due to the nature of the study design, participants are not blinded to the tango treatment itself; however, as the waiting-list control group will as well receive the same tango lessons later on, they will not know whether they are in the waiting list or not. According to their allocation, study participants will be individually requested by the members of the research team to complete the questionnaires. The flow diagram of the study design is depicted in Fig. 1.

#### Procedure for unblinding if needed {17b}

There is no need of unblinding.

### Data collection and management

#### Plans for assessment and collection of outcomes {18a}

The primary outcome is change in CRF measured with the validated CFS-D questionnaire [30]. The secondary outcomes are sleep quality, measured with the Pittsburgh Sleep Quality Index (PSQI) [31], and patient’s HRQL, measured with the European Organization for Research and Treatment of Cancer Questionnaire C30 (EORTC-QLQ-C30) [32].

After obtaining written informed consent and inclusion in this study, sociodemographic and clinical data are surveyed, and questionnaires will be given to participants. All questionnaires will be collected prior to and post-treatment and during follow-up 6, 12, and 24 months after the start of the tango module (Fig. 1).

#### Plans to promote participant retention and complete follow-up {18b}

If there are problems with participation in a tango session, a phone call will be made to find ways to continue attendance. For the follow-up surveys, participants will be contacted by postal mail, by stamped return envelopes enclosed, and by personal telephone calls.

#### Data management {19}

Pseudonymized data will be collected with paper case report forms and paper-based questionnaires. All essential trial documentation will be kept within the Trial Master File. For all collected data, pseudonymized data entry is performed at the study center by members of the research staff [36, 37]. Clinical and demographic data will be retrieved from the patients during the informed consent interview by the study physician.

#### Confidentiality {27}

Data management and processing of the pseudonymized collected data are subject to the General Data Protection Regulation (GDPR). In order to protect the confidentiality, the pseudonymized collected data will be stored for 15 years after the end of this study in a pseudonymized form at the study center of the FIH at the GKHB. The pseudonymized data may only be reviewed by authorized persons of the research team. No third parties will have access to these data.

#### Plans for collection, laboratory evaluation, and storage of biological specimens for genetic or molecular analysis in this trial/future use {33}

No biological specimens will be collected.

## Statistical methods

### Statistical methods for primary and secondary outcomes {20a}

All statistical analyses will be performed using Excel 2010 (Microsoft, USA) and the software R (R version 4.0.5) [38]. Baseline data will be summarized using descriptive statistics. Continuous variables will be described as means with standard deviation and median with interquartile range (IQR), categorical variables will be summarized as frequencies and percentages. Student’s *t*-tests will be applied, and *p*-values < 0.05 are considered to be significant. For the characterization of group differences, Pearson’s chi-squared tests with Yates’ continuity correction and Student’s *t*-tests will be performed. The detection of longitudinal effects will be characterized with Student’s *t*-tests and to identify influencing factors and to address potential sources of bias and potential confounders, and adjusted multivariable linear regression analyses will be performed. In order to yield reliable model results, stepwise regression selections will be performed, and models with high adjusted *R*^2^ will be chosen. According to Cohen’s interpretation [39], *R*^2^ values between 13 and 25% indicate medium, and *R*^2^ values 26% or above indicate high effect sizes. In addition to statistical significance, we will report descriptive statistics and effect size estimates.

### Interim analyses {21b}

No interim analysis will be performed. No unblinded interim results have been and will not be presented anywhere.

### Methods for additional analyses (e.g., subgroup analyses) {20b}

Subgroup analyses will be performed regarding the possible confounders such as concurrent endocrine treatment, concomitant medications for symptom relief, and sport activity. Multilevel modeling can be used for subgroup analyses and to account for confounding variables. Confounding variables (age, body mass index, tumor stage, hormonal status, endocrine treatment, concomitant medication, sport activity, and the respective outcome values at baseline) will be considered. Furthermore, adjusted multivariable linear regression analyses will be performed, to address potential confounders and influencing factors.

### Methods in analysis to handle protocol non-adherence and any statistical methods to handle missing data {20c}

Patients who are unable to participate in the tango treatment or withdraw consent to participate in the study will be excluded and not replaced. If individual sessions cannot be attended, this will be noted, and the surveys will continue as planned. Regarding the evaluation of the questionnaires, we follow the EORTC-QLQ-C30 scoring manual on the calculation and handling of missing data. We do not plan to use imputation when reporting outcome data.

### Plans to give access to the full protocol, participant level-data, and statistical code {31c}

The trial protocol and anonymized data on the group level may be shared with scientists who have medically or scientifically well-founded reasons; data protection according to GDPR and ethics according to ethical approval must be ensured.

### Oversight and monitoring

#### Composition of the coordinating center and trial steering committee {5d}

The trial coordination team meets regularly with the chief investigator, study physicians, tango teacher, and further research team members. The study design, study protocol, data management, and all study-related documents are critically reviewed. The coordinating investigator is responsible for the trial registration, revisions of the study protocol and application/amendments to the ethics committee, and scheduling of regular team meetings. All involved investigators and team members ensure compliance with the study protocol and ensure the follow-up according to the protocol. The steering committee consisting of clinical experts and sponsor’s staff is not directly involved in the study and ensure the scientific quality of the study, the study protocol, and the study report.

#### Composition of the data monitoring committee, its role, and reporting structure {21a}

Not applicable, this trial is not monitored.

#### Adverse event reporting and harms {22}

During each tango session, information on the occurrence of adverse events is regularly retrieved and documented. Adverse events related to the tango treatment during the 6-week sessions will be recorded by the coordinating investigator.

#### Frequency and plans for auditing trial conduct {23}

The trial office monitors the aspects of the study on an ongoing basis, and no audits are planned. The Trial Steering Committee will meet every 5–7 months.

#### Plans for communicating important protocol amendments to relevant parties (e.g., trial participants, ethical committees) {25}

All protocol deviations or modifications will be documented by appropriate updates in due course and according to the national regulations; all substantial protocol amendments will be communicated to the ethics committee of the Medical Association Berlin (Berlin Ethik-Kommission der Ärztekammer Berlin) and German clinical trial register, DRKS.

#### Dissemination plans {31a}

The study results will be presented in conferences and symposia and submitted for publication in relevant medical journals. Portions from this study will also be included in two doctoral theses.

## Discussion

Here, we present a study protocol on the efficacy of a 6-week Argentine tango module for the reduction of CRF symptoms in people after primary breast cancer treatment. A systematic review and meta-analyses revealed that supervised aerobic exercise in particular was statistically more effective than conventional care in improving CRF in breast cancer patients [40]. As has been reported, multimodal supportive strategies such as mind-body therapies including relaxation techniques, stress management, meditation, physical activity [41], and also physical self-management interventions [42] have been established to counteract adverse effects of oncological therapeutics and seem to generate beneficial effects on perceived HRQL including fatigue levels [8, 43]. Some of these interventions have been included in international guideline recommendations [8, 44, 45]. Currently, it is widely accepted that exercise and physical activity are effective to reduce CRF levels [7], and most breast cancer patients are aware of this and also want to be active, but lack the necessary impulses to do so, especially when having fatigue symptoms. In the past, most studies have addressed HRQL of breast cancer patients during their oncology treatments, and more studies are needed that address symptom burden in breast cancer survivors who have successfully completed their oncology treatments [5]. Therefore, we decided to offer a treatment program that can fill this gap, is not too elaborate and strenuous, and can be performed even with physical limitations.

CRF is a multidimensional syndrome, frequently occurring with other neuropsychological symptoms also acting at sleep/wake centers in the brain [46]. Furthermore, it has been observed that CRF frequently occurs alongside and is interrelated with sleep disturbances [47]. Interventions that affect the biorhythm may therefore also influence the mood and well-being, as well as might modulate fatigue symptoms of cancer patients. Dance can be understood as a multimodal treatment based on body awareness, expression, and rhythm, combining both psychotherapeutic therapy and physical activity, and can support emotional, cognitive, and spiritual integration [48]. A recent study reported that ballroom dancing may improve functional exercise capacity and may be associated with a high self-efficacy and active lifestyle [49]. However, no effects of this dance training on fatigue symptoms were observed, but there was a limitation to individuals who had a healthy dance partner and presumably exhibited only mild fatigue symptoms, since data were collected only from participants of active dance classes [49]. To address people with breast cancer who suffer from CRF, have low self-confidence, are less fit, and often do not have any appropriate partner for dance exercises, we chose Argentine tango, a dance style that allows adaptation to different conditions and can be performed even under the current pandemic situation by dancing with a surgical mask and in a distance of 2 m. The therapeutic use of Argentine tango to reduce fatigue symptoms in breast cancer patients has not yet been assessed. Argentine tango comprises relaxation techniques, supports balance finding and self- and spatial perception exercises, and thus may influence physical, psychological, and cognitive skills as well as probably also fatigue levels and insomnia. It is expected that receiving the 6-week tango module will significantly reduce fatigue and insomnia symptoms in our breast cancer patients. Previously, we could show that the treatment of breast cancer patients with chemotherapy is associated with a severe increase in fatigue, in particular, of cognitive fatigue [50]. There are hardly any therapeutic options to alleviate such cognitive impairments. It has been reported that Argentine tango can influence and improve cognitive abilities in Parkinson’s patients [29]. Therefore, it is believed that the tango may also have a positive effect on cognitive fatigue in breast cancer patients. The tango group is expected to have a clinically relevant reduction in fatigue and insomnia symptoms compared to the waitlist control. Further benefits related to aspects of HRQL may be observed. In addition, follow-up surveys of the entire cohort will be conducted at 6, 12, and 24 months to examine how this tango treatment will be perceived by the participants and how lasting the effects prove to be.

## Trial status

The current protocol version is 2.0, dated from 23 March 2020. The trial is ongoing and currently enrolling. The first participant was enrolled on 10 June 2020, and recruitment is expected to be finalized in the second quarter of 2022 with the follow-up completed by the end of March 2024.

## Abbreviations

CRF: Cancer-related fatigue
CFS-D: German version of the Cancer Fatigue Scale
ECOG: Eastern Cooperative Oncology Group
EORTC: European Organisation for Research and Treatment of Cancer
FIH: Forschungsinstitut Havelhöhe
GDPR: General Data Protection Regulation
GKHB: Gemeinschaftskrankenhaus Havelhöhe Berlin
HRQL: Health-related quality of life
PSQI: Pittsburgh Sleep Quality Index
